# Supplementary figures and images for: Understanding the Influence of Patient Factors on Accuracy and Decision-Making in a Diagnostic Accuracy Study with Multiple Raters—A Case Study from Dentistry
Source: Int J Environ Res Public Health. 2023 Jan 18;20(3):1781. doi: 10.3390/ijerph20031781 (PMC9914814; doi:10.3390/ijerph20031781)

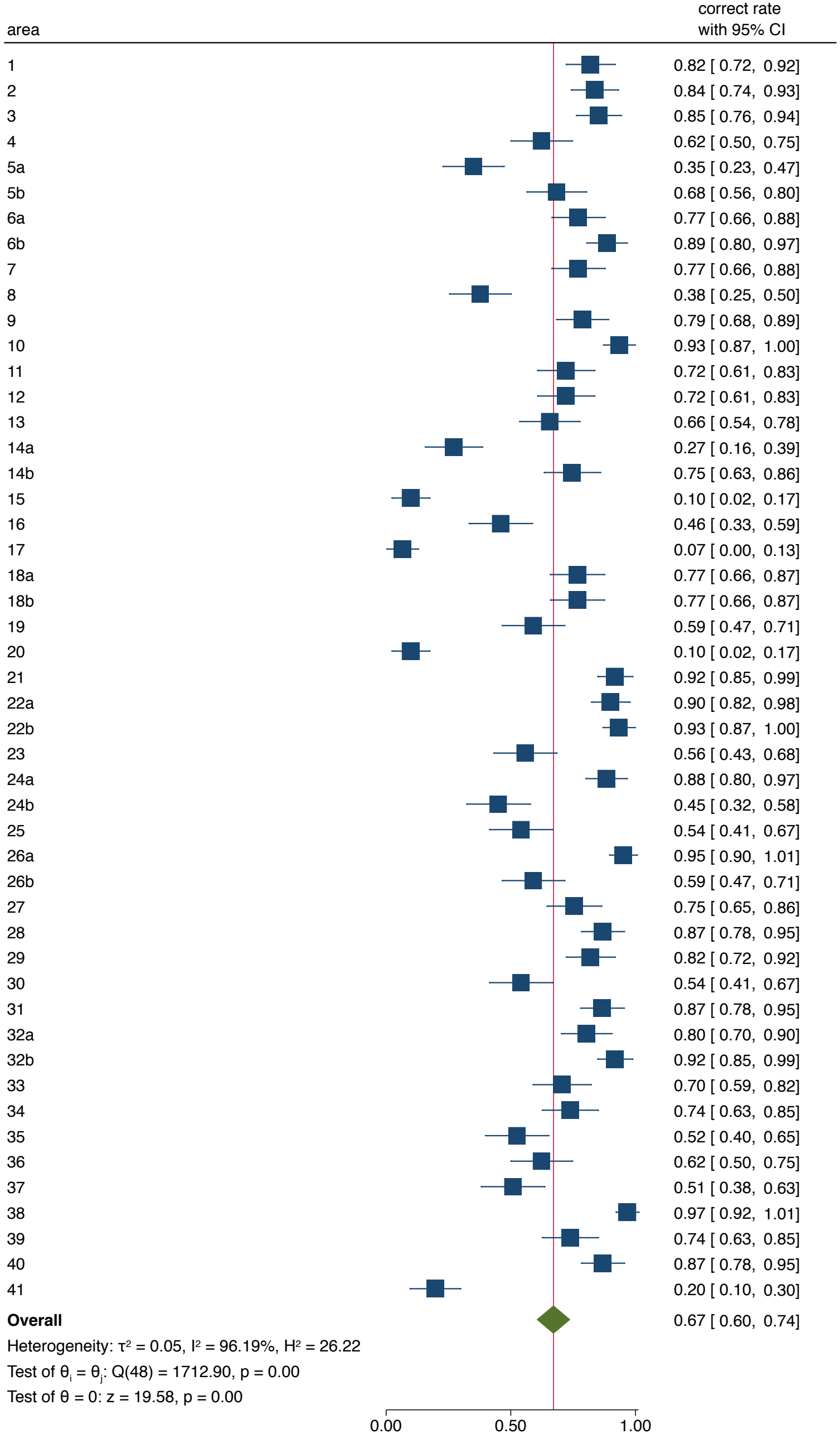

Supplement: Supplementary file 1 [file ijerph-20-01781-s001.zip › FigureS1.pdf]

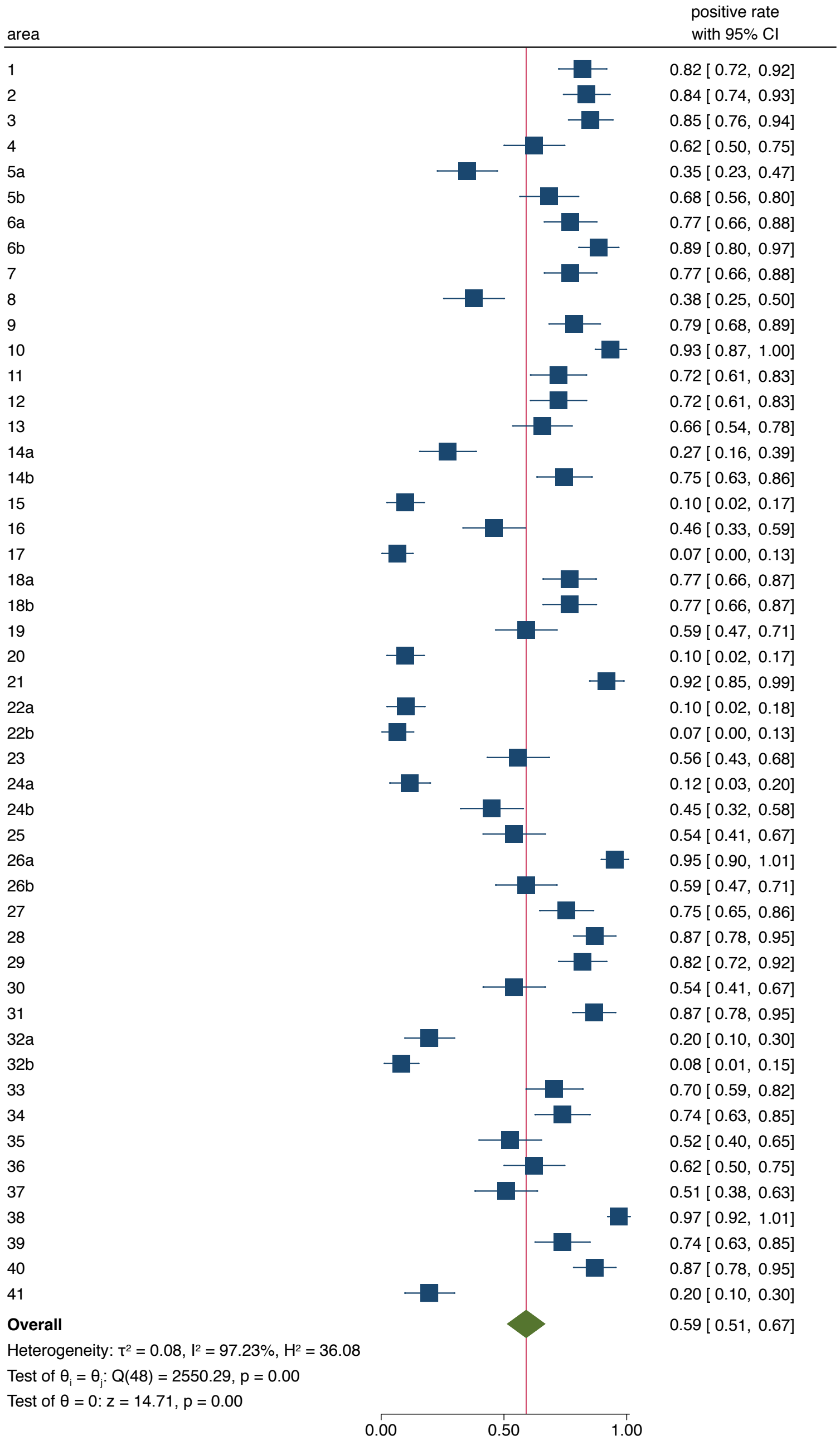

Supplement: Supplementary file 1 [file ijerph-20-01781-s001.zip › FigureS2.pdf]
